# Protective effects of safranal on diabetic retinopathy in human microvascular endothelial cells and related pathways analyzed with transcriptome sequencing

**DOI:** 10.3389/fendo.2022.945446

**Published:** 2022-11-16

**Authors:** Qin Xiao, Yao-Yao Sun, Zhan-Jun Lu, Shan-shan Li, Riguga Su, Wen-Lin Chen, Lin-Lin Ran, Surina Zhang, Kaixin Deng, Wen-Zhen Yu, Wenqian Chen

**Affiliations:** ^1^ Department of Ophthalmology, Affiliated Hospital of Inner Mongolia University for Nationalities, Tongliao, China; ^2^ Department of Ophthalmology, Peking University People’s Hospital, Beijing, China; ^3^ Eye diseases and Optometry Institute, Beijing, China; ^4^ Beijing Key Laboratory of Diagnosis and Therapy of Retinal and Choroid Diseases, Beijing, China; ^5^ College of Optometry, Peking University Health science center, Beijing, China; ^6^ College of Clinical (Mongolian) Medicine, Inner Mongolia University for Nationalities, Tongliao, China; ^7^ Department of Hematology, Affiliated Hospital of Inner Mongolia University for Nationalities, Tongliao, China; ^8^ Department of Ophthalmology, Beijing Jishuitan Hospital, Beijing, China

**Keywords:** diabetic retinopathy, safranal, high glucose, transcriptomics, human retinal microvascular endothelial cells

## Abstract

**Aim:**

To determine the effect of safranal on diabetic retinopathy *in vitro* and its possible mechanisms.

**Methods:**

We used human retinal microvascular endothelial cells (HRMECs) to test the influence of safranal *in vitro*. High glucose damage was established and an safranal was tested at various concentrations for its potential to reduce cell viability using the MTT assay. We also employed apoptosis detection, cell cycle detection, a transwell test, and a tube formation assay to look into safranal’s inhibitory effects on high glucose damage at various doses. Furthermore, mRNA transcriptome sequencing was performed. mRNA expression levels in a high glucose damage model, a high glucose damage model treated with safranal, and a blank control were compared to find the possible signaling pathway. Western blotting was used to confirm the expressions of several molecules and the levels of phosphorylation in each for the newly discovered pathway.

**Results:**

Cell proliferation was inhibited under a high glucose condition but could be protected by safranal at different concentrations (P<0.001). Flow cytometry results suggested safranal also protected cells from apoptosis (P=0.006). A transwell test demonstrated reduced invasiveness of safranal-treated cells in a high glucose condition (P<0.001). In a tube formation investigation, there were noticeably more new branches in the high gloucose group compared to a high glucose treated with safranal group (P<0.001). In mRNA expression patterns on transcriptome sequencing, the MAPK signaling pathway showed an expression ratio. With western blotting, the phosphorylation level of p38-AKT was elevated under a high glucose condition but could be inhibited by safranal. The expression of molecules associated with cell adhesion, including E-cadherin, N-cadherin, Snail, Twist, and fibronectin also changed significantly after safranal treatment under a high glucose condition.

**Conclusion:**

Safranal can protect diabetic retinopathy *in vitro*, and the p38-AKT signaling pathway was found to be involved in the pathogenesis of diabetic retinopathy and could be inhibited by safranal. This pathway may play a role by influencing cell migration and adhesion.

## Introduction

Diabetic retinopathy (DR) is the most prevalent and distinct microvascular diabetic complication. In individuals aged 20 to 74 years, it is still the major cause of vision loss and preventable blindness, especially in middle- and high-income nations (1, 2). According to Zhen et al., in 2020, 103.12 million adults worldwide were forecast to have DR. By 2045, that figure is expected to rise to 160.50 million (3). The number of persons who lose their vision due to DR is likely to continue to climb and will become an even more critical issue in the future.

DR is thought to be a microvascular disease. Through influences on cellular metabolism, signaling, and growth factors, several biochemical processes have been postulated to modulate the pathogenesis of DR (4). Recently, people have investigated the mechanisms of high glucose damage on vascular alterations and DR development (5). As an illustration, it was discovered that high glucose causes the production of inflammatory intermediates, the breaching of the blood-retina barrier, the demise of pericytes, and an increase in vascular permeability, all of which contribute to the progression of DR phases and the development of vascular dysfunctions (6, 7).

Attempts to uncover novel therapeutic targets have been made despite tremendous breakthroughs in the treatment of DR. Antioxidative stress is gaining attention as a potential treatment for DR. A classical antioxidant; i.e., safranal (systematic name 2,6,6-trimethylcyclohexa3,1-din-1-carboxaldehyde), is a compound of volatile carboxaldehyde (8) and a major constituent of saffron. Safranal has a wide spectrum of biological actions, including anti-inflammatory, anti-cancer, and antigenotoxic characteristics (9, 10).. In DR patients, precursors of safranal, such as crocin, have been reported to be utilized to reduce microglial activation (11). It can also reduce central macular thickness and increase best-corrected visual acuity (12). Our previous study revealed an inhibitory function for safranal on chroidal neovascularization. However, the role of safranal in DR and the mechanisms involved have not been well investigated.

This study aimed to ascertain whether safranal was protective for DR *in vitro* and to elucidate the mechanism behind this effect. With human retinal microvascular endothelial cells(HRMECs), we verified the protective effect of safranal on DR under severe glucose damage *in vitro*. Finally, we identified and confirmed the potential pathway involved in using transcriptome sequencing.

## Methods

### Cell viability

Cellular viability of HRMECs was analyzed using an MTT assay (MTT Cell Proliferation and Cytotoxicity Assay Kit; Beyotime, Shanghai, China) according to the manufacturer’s instructions and as previously reported (13). Cells were plated at 5×10^4^ per well in 96-well plates, and cell proliferation was measured. High glucose was added to the wells at different concentrations. 10 μL of safanal were added to create an experimental group and 10 μL of added phosphate-buffered saline (PBS) was used as the blank control. Cells were cultured at 37°C. 10 μL of MTT were added into each well and incubated at 37°C for 4 h and then the absorbance (A) of each well was estimated at 568 nm. Each experiment was repeated in five wells and was duplicated at least three times. Optical density (OD) values were obtained and the inhibition rate was calculated as (1-OD experiment group/OD negative control group) ×100% As previously reported, the MTT assay (MTT Cell Proliferation and Cytotoxicity Assay Kit; Beyotime, Shanghai, China) was used to evaluate the cellular viability of HRMECs. Cell proliferation was assessed after cells were plated in 96-well plates at a density of 5×10^4^ per well. The wells received additions of high glucose at various concentrations. The experimental group received 10 μL of safanal, and the blank control received 10 μL of phosphate-buffered saline (PBS). At 37°C, cells were cultured. Each well received 10 μL of MTT, which was then added, and each well’s absorbance (A) was calculated at 568 nm after being incubated at 37°C for 4 hours. Each experiment was done at least three times in 5 distinct wells. The inhibition rate was estimated as (1-OD experiment group/OD negative control group) 100% using the optical density (OD) values (10, 14).

### Flow cytometric analysis

A FITC Annexin V Apoptosis Detection Kit (BD Biosciences, San Diego, CA) was used to quantify apoptosis in accordance with the manufacturer’s recommendations. In summary, HRMECs (1×10^6^) were plated in six-well plates and exposed to high glucose, high glucose with safranal, or a control solution for 24, 48, or 72 hours. Following EDTA-mediated cell detachment, the cells were washed in cold PBS and stained with annexin V-FITC and propidium iodide (PI). Flow cytometry analysis was carried out immediately (excitation 488 nm; emission 530 nm). CellQuest software was used to analyze the samples using flow cytometry (FACSCalibur; BD Biosciences) (BD Biosciences). The percentage of early apoptotic cells (LR) plus late apoptotic cells was used to compute the apoptotic rate (UR) (15). The CycleTESTTM Plus DNA Reagent Kit (BD Biosciences) was used to stain cells with PI for cell cycle analysis, and FACScan was used to analyze the results. Cells in the G0/G1, S, and G2/M stages were enumerated, and their percentages were compared.

### Migration

As previously mentioned (14, 15), migration was evaluated using a transwell (Corning Life Sciences, Lowell, MA) with an 8.0 m pore size. Briefly, 2×10^4^ cells in 200 μL of serum-free media were added to the transwell’s top. A final amount of 600 μL of DMEM (with 10% FBS) was added to the bottom chamber. Every migration test was run for 5 hours at 37°C. The cells were stained with 4,6-diamidino-2- phenylindole (DAPI; Roche Diagnostics, Indianapolis, IN) for 15 minutes after the assay and then fixed in 4% paraformaldehyde. A cotton swab was used to remove the immobile cells so the membrane could be photographed. Five arbitrary fields of view’s worth of cells were counted.

### Tube formation

Cells were grown with high glucose, high glucose plus safranal, or a negative control solution at a density of 2×10^5^ cells per well. According to the manufacturer’s directions and our earlier research, 48-well plates were filled with 150 μL of matrigel (Cat# 354234; BD Sciences, Franklin Lakes, NJ) solution and incubated at 37°C for 0.5 h. The matrigel was seeded with HRMECs (5×10^4^ per well), which were then cultivated for 2–8 hours. Matrigel networks were counted and taken pictures of. Image J software was used to count the new branches and branch nodes (National Institutes of Health, Bethesda, MD). Three times the experiments were conducted (10, 16).

### Transcriptome sequencing

Three groups were created after counting the cells: Negative control, high glucose damage model treated with high glucose (10 μL, 25 mol/L), and high glucose damage model treated with safranal (10 μL, 80 g/mL) are the three options. As previously mentioned, cells were cultured. As previously reported (10, 16), RNA samples were isolated and identified. With the use of oligo(dT)-coupled magnetic beads, mRNA was amplified; random hexamers were used to create single-stranded cDNA; and buffer, dNTPs, DNA polymerase I, and RNase H were used to create double-stranded cDNA. The end of the purified double-stranded cDNA was first repaired, then an A tail addition was added, the sequencing connector was connected, and finally the fragment size was chosen using AMPure XP beads.The final step was performing PCR amplification and purifying the PCR output with AMPure XP beads to create the final library. Different libraries were pooled into the flow cell in accordance with the effective concentration and the demands of the target dismount data volume after the library detection was qualified. Following cBOT clustering, sequencing was carried out using an Illumina high-throughput sequencing platform.

### Western blotting

HRMECs were counted and separated into three groups: a negative control; a high glucose damage model treated with safranal (10 μL, 80 g/mL); and a high glucose damage model treated with high glucose (10 μL, 25 mol/L). A Bio-Rad test kit was used to extract the cells’ total protein and evaluate the protein content (Bio-Rad, Hercules, CA, USA). Equal amounts of protein (30 μg) were resolved on polyacrylamide gels containing 12% Tris-HCl before being transferred to a PVDF blotting membrane (Millipore, Billerica, MA, USA). After blocking, specific antibodies against phosphate-extracellular regulated protein kinases (p-ERK), total-ERK (t-ERK), p-P38, t-P38, p-serine/threonine kinase (AKT), t-AKT (1:2000, Santa Cruz, CA, USA), E-cadherin, N-cadherin, Snail, Twist, Fibronectin(1:2000, Santa Cruz, CA, USA) and beta actin (1:2,000, Abcam, Cambridge, MA, USA), were treated with the membranes. The protein bands were detected by chemiluminescence after the blots had been carefully washed and treated with peroxidase-conjugated goat anti-rabbit or anti-mouse secondary antibodies (1:1,000, ZSGB-Bio, Beijing, China) (Pierce, Rockford, IL, USA). Three times the experiment was conducted (10).

### Statistical analysis

Each of the tests were conducted three times, and the data are shown as the mean standard error of the mean (SEM). A one-way ANOVA was used for three groups or more and the Student’s t test was used for two groups. Statistics were considered significant if P<0.05. Using SPSS 17.0, all data analysis were carried out (Chicago, IL, USA).

## Results

### Safranal shows a protective effect of cell proliferation on high glucose damage in HRMECs

**Figure 1:**
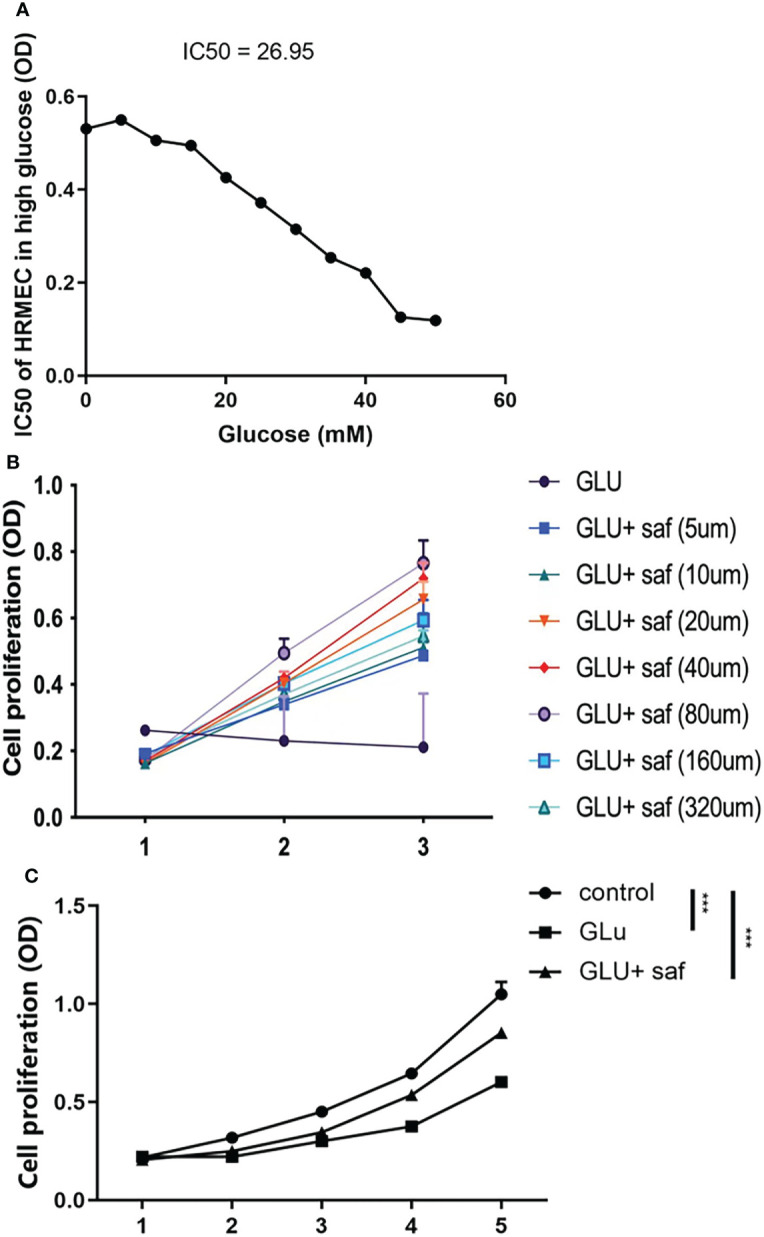
Safranal’s protective effect on cell proliferation in HRMECs in high glucose damage conditions. **(A)** shows that under different concentrations of glucose, cell proliferation was inhibited with an IC50 of 26.95. Cell proliferation was protected under different concentration of safranal while 80 μM demonstrated the best protection effect **(B)**. After five days of incubation, safranal had a better protective effect on cell proliferation over time P<0.001; **(C)**.

In order to determine the appropriate glucose concentration for construction of the high glucose damage model, we investigated the effect of different concentrations of glucose on cells of 0 μM, 5 μM, 10 μM, 15 μM, 20 μM, 25 μM, 30 μM, 35 μM, 40 μM, 45 μM, and 50 μM. We found a dose-dependent effect on cell proliferation, where the IC50 of glucose was 26.95 (**Figure 1A**). Combining our result with previous report, we chose 25 μM as the concentration for subsequent experiments, we chose 25 μM as the concentration for subsequent experiments (17). At a high glucose concentration of 25 μM, we screened different safranal concentration gradients (5 μM, 10 μM, 20 μM, 40 μM, 80 μM, 160 μM, and 320 μM). After three days of cell culture, we found a similar dose-dependent effect of safranal on cell proliferation. Although high glucose inhibited cell proliferation, this inhibitory effect was ameliorated with higher safranal doses. When the concentration exceeded 160 μM, however, there was an effect of toxicity (**Figure 1B**). Therefore, we chose 80 μM as the final concentration for further study. We further investigated cell proliferation, and after five days of co-culture, we found that, cell growth was decreased in the presence of high glucose damage, but safranal exhibited a protective impact in comparison to the control, and this protective effect became more pronounced with longer safranal incubation time. (P<0.001; **Figure 1C**).

### Safranal protects HRMECs from apoptosis and affects the cell cycle under a high glucose condition and *in vitro*

**Figure 2:**
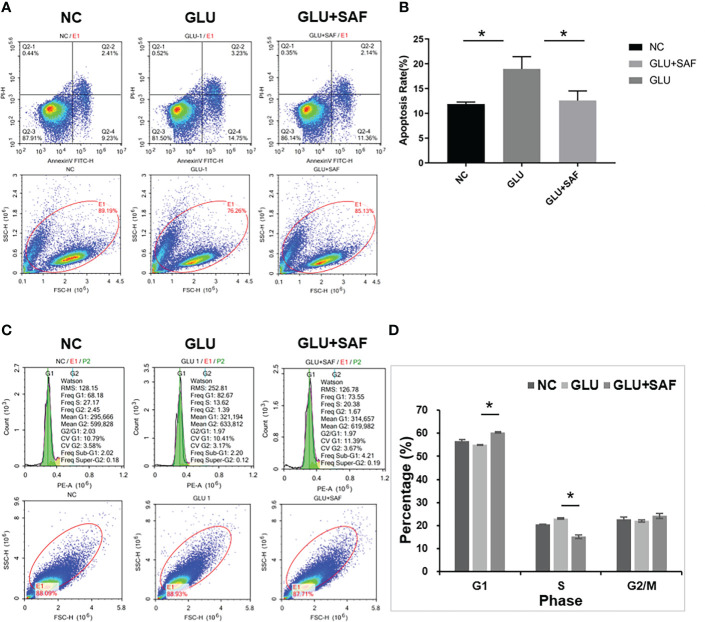
Flow cytometry results. The apoptosis rate varied indifferent groups:negative control, high glucose group, and high glucose group treated with safranal P=0.006, **(A, B)**. Regarding the cell cycle, compared with the high glucose group, there were more cells in phase G1 but fewer cells in phase S in the high glucose treated with safranal group P<0.001; **(C, D)**. * means a statistically significant difference.

The effects of safranal on apoptosis in HRMECs was assessed by A FITC Annexin V Apoptosis Detection Kit. As shown in **Figures 2A, B**, under high sugar conditions, more cells underwent apoptosis. However, this apoptosis was inhibited after the treatment with safranal. Under a high glucose condition, HRMECs treated with safranal showed a significantly lower percentage of apoptotic cells (P=0.006).

Next, we examined the effect of safranal on the cell cycle G1 or S phase by using flow cytometry analysis. From **Figures 2C, D**, we found that high glucose reduced the cell number in phase G1, while the number in phase S were increased. After safranal treatment, however, we found more cells in the G1 phase and fewer cells in phase S (P<0.001). These results suggested that safranal could inhibit cell apoptosis and affect the cell cycle.

### Safranal inhibits cell migration of HRMECs *in vitro*

**Figure 3:**
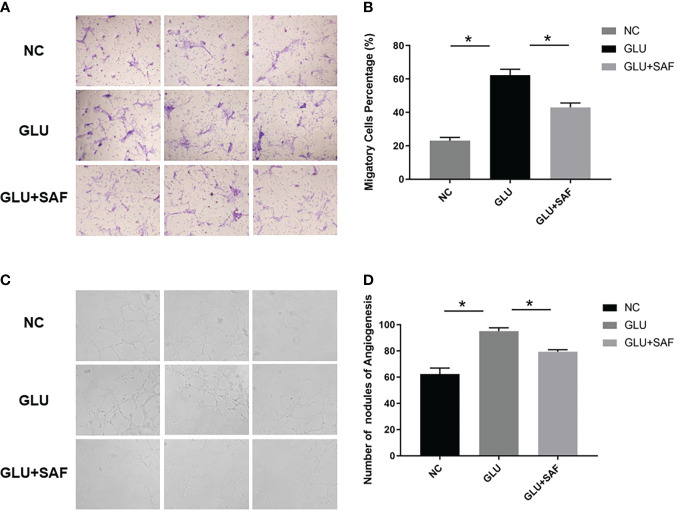
Cell migration and tube formation results. Under high glucose, more cells were found to migrate through the membrane. After safranal treatment, the migration rate decreased significantly P<0.001, **(A, B)**. There were more new vessel branches and nodes in the high glucose group but both decreased after safranal treatment P<0.001, **(C, D)**. * means a statistically significant difference.

Along with the cell proliferation investigation, we also investigated into whether safranal affects HRMEC migration, a critical stage in the creation of vessels. The modified Boyden chamber test was used. According to **Figures 3A, B**, more cells went through the membrane under the high glucose condition than they did under the negative control. Cellular migration was, however, inhibited by safranal. The high glucose group and the high glucose group treated with safranal differed significantly. (P<0.001; **Figures 3A, B**).

### Safranal inhibits tube formation of HRMECs *in vitro*

In the *in vitro* tube formation investigation, higher tube development was seen when the glucose level was high. However, the 80 μM safranal-cultured HRMECs showed a reduced ability to develop a normal vascular network. In comparison to the control groups, the network’s number of new branches and branch nodes dramatically dropped. (P<0.001; **Figures 3C, D**).

### Pathways involved in cellular high glucose damage and the effect of safranal identified with transcriptome sequencing

**Figure 4:**
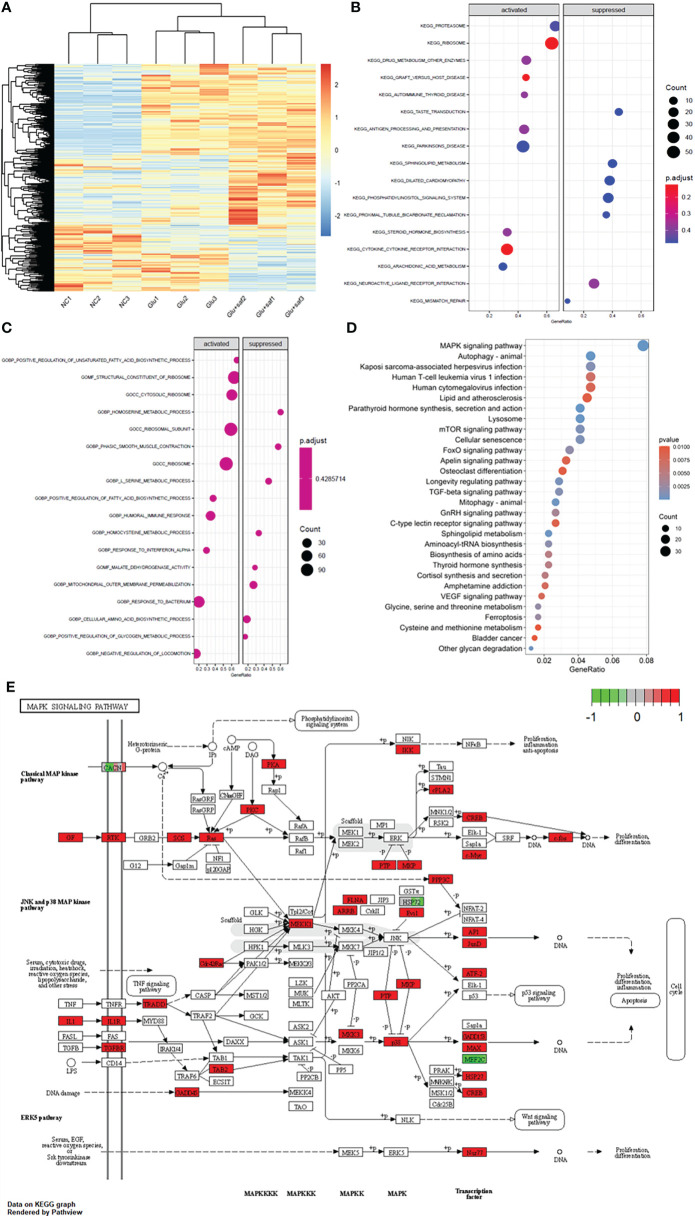
Transcriptome sequencing results. **(A)** shows the heat map of the three groups including the negative control, high glucose group, and high glucose treated with safranal group. **(B, C)** demonstrate the Kyoto Encyclopedia of Genes and Genomes (KEGG) and Gene Ontology (GO) results between the high glucose group and high glucose treated with safranal group. From the bubble graph, the MAPK signaling pathway demostrateda high gene ratio **(D)** and the detailed pathway was demonstrated in **(E)**.

**Figure 4A** shows the heat map of the three groups, including the negative control, high glucose group, and high glucose treated with the safranal group. **Figures 4B, C** demonstrate the Kyoto Encyclopedia of Genes and Genomes (KEGG) and Gene Ontology (GO) results between the high glucose group and high glucose treated with the safranal group. From the KEGG results for these two groups, after safranal treatment, the ribosome pathway, Parkinson’s disease-related pathway, and cytokine receptor interaction pathways are the major activation pathways. In contrast, the dilated cardiomyophaty pathway and phosphatidylinositol signalings system pathway are the major inhibition pathways. From the GO results for these two groups, after safranal treatment, the ribosome pathway, structural constituent of the ribosome pathway, ribosomal subunit pathway, and response to bacterium were the major activation pathways, while mitochondrial outer membrane permeabilization, the cellular amino acid biosynthetic process pathway, and the serine metabolic process pathway were the major inhibition pathways. After analyzing the data from both the KEGG results and GO results, the mitogen-activated protein kinase (MAPK) signaling pathway was activated in the high glucose group and suppressed in the safranal-treated group; it is suggested that safranal treatment DR through this signaling pathway (**Figure 4D**). **Figure 4E** demonstrates the changes in the specific and detailed signaling of the MAPK pathways from KEGG and GO results.

### P38-AKT signaling pathway confirmed by western blotting

**Figure 5:**
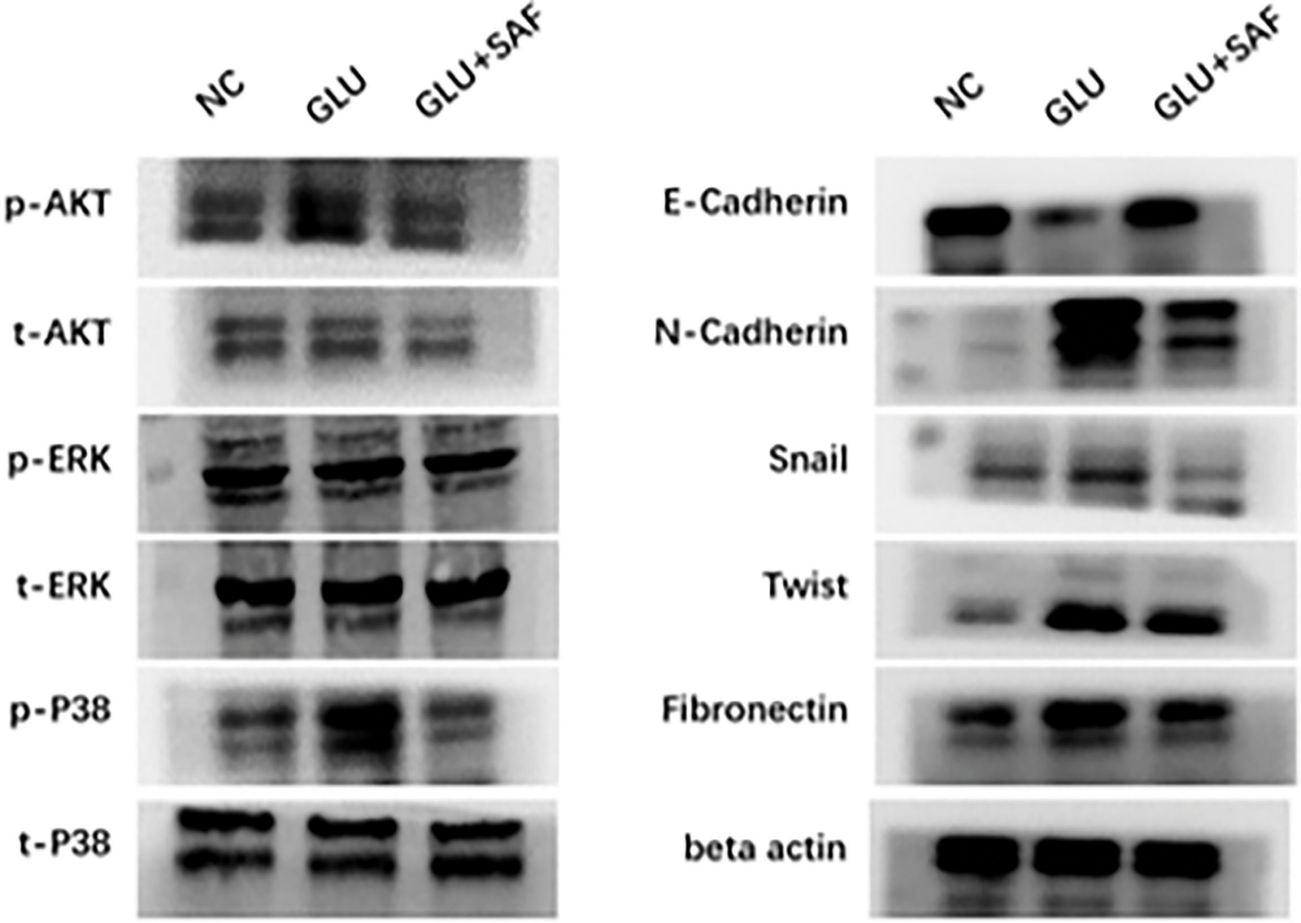
Western blot results. Western blot results showing the phosphorylation changes in the p38-AKT signaling pathway. The phosphorylation level of p38 was elevated in the high glucose condition but was inhibited by safranal. However, there was no change in the phosphorylation level of ERK. Under a high glucose condition, the protein expression of factors related to cell migration and adhesion, including N-cadherin, Snail, Twist, and fibronectin were upregulated in a high glucose condition and could be inhibited by safranal. E-cadherin, on the other hand, demonstrated an opposite expression trend.

Western blot analysis was used to confirm the MAPK members, including P38 and the ERK-related pathway, that transcriptome sequencing had identified. Basically, western blot analysis of p-P38, t-P38, p-ERK, t-ERK, p-PDK1/2, p-AKT, and t-AKT was carried out, with beta actin serving as an internal loading control. We found that under a high glucose condition or after safranal treatment, there was no difference in protein expressions for both p-ERK and t-ERK. On the other hand, the phosphorylation levels of p38 and AKT were elevated in the high glucose group compared with the levels in the negative control group. Under safranal treatment, however, the phosphorylation levels of P38 and AKT were downregulated. As a result, p38-AKT but not the ERK pathway was shown to be possibly involved in the protective effect of safranal on DR. However, further experiments are still needed to explore the intracellular mechanisms (**Figure 5**).

### E-cadherin, N-cadherin, snail, twist, and fibronectin were found to involve in the protection of DR by safranal

Factors related to cell adhesion and migration, including E-cadherin, N-cadherin, Snail, Twist, and fibronectin were also analyzed. Under a high glucose condition, the protein expressions of all these factors were upregulated, except for E-cadherin, demonstrating that high glucose could promote cell adhesion and migration. After safranal treatment, however, the protein expression was decreased for all the factors investigated, except for E-cadherin. E-cadherin demonstrated a total reversed change in that the expression was decreased under a high glucose condition but was increased after safranal treatment. These results indicated that safranal inhibits DR mainly by inhibiting cell adhesion and migration in the presence of high glucose.

## Discussion

In the present research, we demonstrated that safranal had a protective effect on DR *in vitro*. The function of safranal was verified in cells under hyper-glucose conditions. Transcriptome sequencing has been used to further investigate the potential signaling pathway, and it revealed the P38-AKT pathway as being connected to hyper-glucose damage in cells, with the potential for safranal to block this effect. Furthermore, this pathway mainly affects the adhesion and migration-related molecules of cells. Our results confirm that safranal may have a protective effect against DR *in vitro* and that the P38-AKT pathway may be involved. This is the first report on this pathway having a protective effect with safranal treatment for DR.

There have been many attempts to explore ways to slow the progression of DR to the proliferative stage. Many drugs are still being studied, and their preventive or therapeutic properties need to be investigated further. Pharmaceutical investigations have revealed saffron’s component; i.e., safranal, which possesses antioxidant, anti-apoptotic, and anti-inflammatory properties (10). Safranal decreased caspase-3, tumor necrosis factor-alpha (TNF-), or superoxide dismutase (SOD) activity in diabetic rats, according to Malekzadeh et al. (18). In our study, the protective influence of safranal on DR was verified. Safranal could protect cells from the effects of high glucose on cell proliferation, reduce apoptosis, and inhibit cell migration and tube formation, similar to the previous report.

To determine the possible mechanism of how safranal protects DR, transcriptome sequencing was performed to learn more about how safranal functions in cells under high glucose circumstances. The P38-AKT signaling pathway was discovered to be involved in the hyper-glucose injury process in cells after Western blotting confirmed it. Additionally, safranal has the potential to block this signaling pathway. The mitogen-activated protein kinase (MAPK)/p38 signaling pathway is widely known for playing a pivotal role in cell survival and death (19, 20). Targeting the p38 signaling pathway has been shown to influence pathological angiogenesis and tissue repair in other investigations (21). Thus far, research had been conducted on the relationship between P38 and DR. In DR, for example, Zou et al. discovered that p38 might increase retinal micro-angiogenesis by upregulating RUNX1 expression. The p38-MAPK pathway was also found to be activated in diabetic rat model retinopathy with microvascular disease (22). These results confirm our findings, which show that the p38 signaling pathway contributes to the development of DR and that safranal effectively inhibits p38 expression in a model of cellular damage brought on by excess glucose, thus avoiding DR.

When p38 is activated, it causes a cascade of events in downstream signaling pathways, including the phosphorylation of AKT. Cell proliferation, migration, adhesion, apoptosis, and angiogenesis are all regulated by the conventional AKT signaling pathway. In ophthalmological research, the AKT pathway has also been linked to the onset and progression of neovascularization (23, 24). For example, changes in p38 and AKT have been widely shown to be involved in the pathology of DR. However, the ability of the pathogenic effect of this pathway on DR to be protected by safranal is the first report. In our study, however, molecules associated with cell migration and adhesion, including E-cadherin, N-cadherin, Snail, Twist, and fibronectin were also found to participate in safranal protection against DR, so we suggest that changes in AKT mainly affect cell adhesion and migration and thus DR formation. These factors ultimately affect the formation of neovascularization in DR by participating in the migration and adhesion of vascular endothelium cells. When exposed to high glucose, Zhou et al. found that the expression of Snail and N-cadherin levels were increased in Müller cells, which suggested those mesenchymal proteins play an important role in the development of DR. E-cadherin, on the other hand, demonstrated a total opposite expression trend. E-cadherin is closely related to the tight junction of vascular endothelium cells and inner blood-retinal barrier (25). Under the condition of high glucose, the decrease in E-cadherin expression indicates the potential destruction of iBRB (26), while safranal increased the expression of E-cadherin, thus protecting iBRB. Further research is necessary to determine whether safranal’s intervention in DR also involves these other signaling pathways.

This study was unable to determine whether safranal’s effect on DR is directly related to suppression of the P38-AKT pathway. More research is needed to determine the significance of this signaling pathway in safranal’s protection against DR. In addition, other potential DR pathways, such as the VEGF signaling system, were not examined in this study. Further research on the pathophysiology of DR and potential treatment targets is required. Another limitation of the present experiment is that we only performed *in vitro* experiments but not *in vivo* experiments. In future, we need to conduct further *in vivo* experiments to further validate the protective effect of safranal on DR and related pathways.

## Data availability statement

The data presented in the study are deposited in the link as https://datadryad.org/stash/share/K2mdS4ReytQYjgRa5sT-C_4OVs7bF3aKv46rjcvibEM.

## Author contributions

All the authors contributed to Conceptualization, Methodology, Software, Investigation, Formal Analysis, Writing - Original Draft. SL has helped with the data analysis and writing of the paper. All authors contributed to the article and approved the submitted version.

## Funding

This study was supported by the National Natural Science Foundation of China (No.82160815); Inner Mongolia Autonomous Region Science and Technology Project(NO.2022YFSH0061);Research Project of Affiliated Hospital of Inner Mongolia University for Nationalities(NO.KJGGJC202108)

## Conflict of interest

The authors declare that the research was conducted in the absence of any commercial or financial relationships that could be construed as a potential conflict of interest.

## Publisher’s note

All claims expressed in this article are solely those of the authors and do not necessarily represent those of their affiliated organizations, or those of the publisher, the editors and the reviewers. Any product that may be evaluated in this article, or claim that may be made by its manufacturer, is not guaranteed or endorsed by the publisher.
